# Regulatory Reforms for Health Facilities: Can These Suffice? Comment on "What Lies Behind Successful Regulation? A Qualitative Evaluation of Pilot Implementation of Kenya’s Health Facility Inspection Reforms"

**DOI:** 10.34172/ijhpm.2023.7265

**Published:** 2023-03-01

**Authors:** Arthur Bagonza, David Musoke, Henry Wamani

**Affiliations:** ^1^Department of Community Health and Behavioral Sciences, School of Public Health, Makerere University, Kampala, Uganda; ^2^Department of Disease Control and Environmental Health, School of Public Health, Makerere University, Kampala, Uganda

**Keywords:** Regulation, Health Facilities, Low- and Middle-Income Countries

## Abstract

In their study on how Joint Health Inspections (JHI) were implemented in practice with a need to identify key facilitators or barriers for regulatory policy and practice, Tama et al found that innovative regulatory reforms markedly improved inspection scores among intervention health facilities albeit with challenges. Their article makes an important contribution to the body of knowledge in as far as regulation of health facilities is concerned. In low- and middle-income countries (LMICs), private health facilities are poorly regulated and yet, they purge gaps where public health facilities are inadequate as was demonstrated during the COVID-19 pandemic. Therefore, while regulation of public health facilities is standardized, the research by Tama and colleagues provides a unique opportunity to continue dialogue on how private health facilities can be regulated through inspection and supervision. Regulation of public and private health facilities continues to be contentious since both experience unique contextual challenges.

 In their recent article, Tama et al explored the facilitators and barriers to Joint Health Inspections (JHI) of health facilities in a low-income setting. While they found that control of funds was the main barrier in public health facilities, numerous challenges abounded in private health facilities.^1^

 Their article contributes to the way we understand challenges of regulating the health sector which has multiple service providers in low- and middle-income countries (LMICs). Joint facility inspection using a refined tool from all eight regulatory agencies, categorizing health facilities based on an agreed upon criteria and using a randomized controlled trial study design were plausible ways of testing the new innovation. Also, involving a wide range of stakeholders in the implementation process and having an advisory and governance board that are described in the original article were commendable methods of testing whether the JHI that were carried out as a means of reforming regulation of health facilities in LMICs were effective. While the study findings elucidated in the article are extremely important, we need to continue thinking in a broader sense how we can further improve the field of health facility regulation in general and inspection in particular. We need to find out: what more can we do to reduce the hostility between inspectors and private health providers? How can we improve the licensing process of health facilities? And most pressingly, how do we get policy-makers and other government technocrats to implement evidence generated from such studies even though such evidence may have far reaching consequences?

## Inspection, Regulation and Beyond

 Among their study findings, the authors documented that staff working in health facilities felt that the JHI were fair. This finding was due to the fact that a copy of the JHI checklist and a summary report outlining deficiencies and potential areas for improvement was availed. These findings may have accrued as a result of the specific instructions given to inspectors during training. Results in the article are well synthesized from verbatim scripts of respondents, and the methodologies used to arrive at the results are well articulated. However, since there is no standard curriculum or manual for training inspectors approved by the World Health Organization (WHO), we are not in position to state with utmost certainty what inspection styles work in the different contexts.

 None the less, we keep advocating for good inspection practices. Given that good inspection practices lead to better adherence to set statutory guidelines resulting in better quality of care, it is important to continue thinking about how best we can improve inspection of health facilities.

 In the past fifteen years, there has been a marked interest in healthcare provision especially among private healthcare providers in LMICs. This is because many people go to private health facilities to get the first form of medical care. This situation coupled with the chronic brain drain by developed countries, inadequate funding of public health facilities by governments, and high population growth that does not match public health facility carrying capacity implies that private health providers can no longer be ignored. As such, a huge interest has developed on how best a sector with both private and public health providers can be regulated through inspection using evidence-based policies.

 Scholars studying regulation of the health sector are still trying to understand the different approaches and styles which can translate generated evidence into action because evaluation of the current approaches still falls short of producing a standard. None the less, efforts by researchers and scientists to generate evidence that should inform decisions is commendable. However, the procedures used to generate this evidence are neither backed by solid evidence nor appraised.

 Despite the fact that standard techniques to evaluate regulatory processes remain broadly under developed, an evidence-based policy-making tool has been developed by the Economic Commission of the West African States together with the West African Health Organization.^2^ It is believed that with this guidance, an intervention such as that of Tama and colleagues should be able to answer such questions as to whether the intervention produces the expected regulatory reforms and whether it contributes to health systems strengthening.

 Tama and colleagues’ study is a good evaluation of regulatory reforms in general and inspection in particular which builds on work by other scholars that have attempted to evaluate regulatory processes in the health sector.^3^ However, as we gather more evidence on acceptable ways of regulating a complex mix of health service providers, in the opinion of the authors of the regulatory reforms study, something can be done about curtailing the free flow of funds in the public sector. However, in the private sector, the plethora of challenges leaves one main question unanswered – can regulatory reforms suffice? Perhaps this may be the right time to think about implementing multi-sectoral interventions that support regulatory reforms.

## Regulatory Reforms’ Unrealized Possibilities

 Regulation of the health sector faces two main challenges. First and foremost, in many LMICs, staff in health facilities expect inspectors charged with making sure health facilities comply with statutory regulations to also be involved in support supervision. While inspection of health facilities in many LMICs is typified by fault finding with intention to institute punitive measures to errant operators, support supervision focuses on improving performance and building relationships. Indeed, that is why in the study by Tama and colleagues, compliance scores in the intervention facilities improved compared to the comparison facilities. More so, there was a marked improvement in private compared to public health facilities. This finding may have been due to the fact that compliance was underpinned by a personal benefit that was likely to accrue and the procedural fairness used by the joint inspectors. However, the article does not describe how best the current unacceptable reality can be addressed.

 Secondly, Tama and colleagues’ article contributes to understanding the importance of having regulatory reforms being anchored and monitored by an agreed upon consortium. Yet, despite all the existing evidence and structures, regulatory reforms do not reflect the cost and potential advantages they can translate into to achieve the desired patient outcomes. As scholars, we cannot help but ask why and what else needs to be done for such work to be properly embraced by the wider health system actors?

 The health system actors were challenged by the COVID-19 pandemic which overstretched public health facilities, forcing attention to be given to the ever-neglected private health providers in LMICs. This should have been seen as an opportunity to re-build health systems with private sector health providers being more included using policy-backed evidence such as that provided by Tama and colleagues. Even though challenges and limits abound to how much evidence can be extracted for health system strengthening, lessons can be learned from other disciplines such as smart manufacturing and architecture.^4,5^ Indeed, we do not need to reinvent the wheel but rather understand how other disciplines which are not necessarily health related have succeeded with inspection. One thing to note is that we need to remain focused and not get lost in the vast evidence that may not be contextually applicable to health.

## Inclining Our Thinking to Where Evidence May Be Most Needed

 Ideally, all health policies should be backed by evidence. However, a huge gap often exists between the ideal and what actually takes place. This is because in many LMICs, policy-making is sometimes based on the interests of foreign health development partners on one hand and a few elites who may include Ministry of Health officials and other stakeholders with vested interests – all of whom are likely barriers to achieving the full potential of regulatory reforms.^6^

 In the process of implementing regulatory reforms, one may need to ask how financial transparency especially for human resources for health can be achieved. The World Bank Group together with the Bill and Melinda Gates Foundation have piloted the use of digital payments to address this impasse.^7^ However, the trials have started with health workers in public health facilities and yet, public health facilities equally have their unique challenges. Therefore, while the digital payments intervention may aid in streamlining regulatory reforms in the public sector, a lot of skepticism abounds in the private sector. It should also be noted that while scientists struggle to produce evidence that can be used for streamlining the private sector, forces of demand and supply sometimes surpass the need for evidence.^8^ For example, during the recent lockdowns due to the ongoing pandemic, many high level public health facilities that were traditionally the last place for referral were reserved for critically ill COVID-19 patients. As such, patients with ailments other than COVID-19 resorted to private health facilities many of which had to adapt to handle the crisis that was at hand. Therefore, while the best practice would have been to provide evidence on whether these private health facilities were equal to the task followed by appropriate inspection for adherence to statutory guidelines, saving lives was paramount. Such scenarios leave several unanswered questions such as: should regulatory reforms be implemented only when it is convenient or even when times are tough?

 In addition, many LMICs have a list of diseases that are of major concern to adults and children. For this reason, scientists should pay keen interest while producing evidence where regulatory reforms backed by evidence may be needed and ready for sharing with policy-makers.^9^

 It would therefore be inappropriate to think that regulatory reforms will be a silver bullet that will address all compliance issues in the health sector. Instead, scientists should consider taking a closer look at other facets that have helped to improve strengthen compliance in health service delivery. These include but are not limited to: task shifting such that lower level cadre acquire skills similar to those of senior health specialists as a way of policy and regulatory support.^10^ Franchising of health services has also been documented to align with compliance since the outlets operate under one approved license and offer similar health services to a wider population.^11^

## Future Prospects

 By understanding the disease burden by age group, it is possible that LMICs can mobilize implementing partners and focus resources that will aid regulators in issuing the appropriate evidence-based directives to health actors in a way that is socially acceptable. This approach is envisaged to lead to better adherence to statutory guidelines hence improve the quality of care in these countries. Some LMICs have already formed alliances with high income nations to form consortia such as the Child Health Task force which is charged with dissemination of research evidence to stakeholders who include but are not limited to policy-makers.^12^ It is therefore imperative that a strong linkage be formed between researchers and policy-makers where an honest and open discussion can take place to find solutions to the most pressing needs.^13^ This collaboration will improve the uptake of evidence while minimizing the wastage of scarce resources thus quickening and streamlining the policy decision making process. However, we must also exercise extreme caution when evaluating innovations aimed at supporting regulatory reforms for the health sector. That is the only way we shall be able to clearly understand which strategies deserve re-capitalization and eventual scaling up before re-deployment. It is our hope that as scholars engage more with policy-makers, evidence-led policies will be at the fore front of sound policy-making.

## Acknowledgements

 Special thanks go to Dr. Henry Wamani who retires after contributing greatly to generating knowledge in private sector work. May the almighty God bless your onward journey.

## Ethical issues

 Not applicable.

## Competing interests

 Authors declare that they have no competing interests.

## Authors’ contributions

 All authors contributed equally to this paper.
